# Ion channels in lung cancer: biological and clinical relevance

**DOI:** 10.3389/fphar.2023.1283623

**Published:** 2023-10-24

**Authors:** Chiara Capitani, Ginevra Chioccioli Altadonna, Michele Santillo, Elena Lastraioli

**Affiliations:** ^1^ General Pathology Laboratory, Department of Experimental and Clinical Medicine, Internal Medicine Section, University of Florence, Florence, Italy; ^2^ Department of Medical Biotechnologies, University of Siena, Siena, Italy

**Keywords:** lung cancer, SCLC, NSCLC, potassium channels, sodium channels, calcium channels, chloride channels, anion channels

## Abstract

Despite improvements in treatment, lung cancer is still a major health problem worldwide. Among lung cancer subtypes, the most frequent is represented by adenocarcinoma (belonging to the Non-Small Cell Lung Cancer class) although the most challenging and harder to treat is represented by Small Cell Lung Cancer, that occurs at lower frequency but has the worst prognosis. For these reasons, the standard of care for these patients is represented by a combination of surgery, radiation therapy and chemotherapy. In this view, searching for novel biomarkers that might help both in diagnosis and therapy is mandatory. In the last 30 years it was demonstrated that different families of ion channels are overexpressed in both lung cancer cell lines and primary tumours. The altered ion channel profile may be advantageous for diagnostic and therapeutic purposes since most of them are localised on the plasma membrane thus their detection is quite easy, as well as their block with specific drugs and antibodies. This review focuses on ion channels (Potassium, Sodium, Calcium, Chloride, Anion and Nicotinic Acetylcholine receptors) in lung cancer (both Non-Small Cell Lung Cancer and Small Cell Lung Cancer) and recapitulate the up-to-date knowledge about their role and clinical relevance for a potential use in the clinical setting, for lung cancer diagnosis and therapy.

## 1 Lung cancer epidemiology

According to the most recent estimates, lung cancer (LC) is still the most lethal cancer worldwide, responsible of 1.8 million deaths, and the second most frequent in both sexes, with 2.2 million new cases diagnosed in 2020 (Source: Globocan, https://gco.iarc.fr accessed on 10 August 2023). LC is more frequent in high-income countries, mainly due to smoking habits that represent the main risk factor for this malignancy (McIntyre and Ganti, 2017; Sung et al., 2021). Other common risk factors are the exposure to some chemicals such as asbestos (Markowitz, 2022), mustard gas (Ghanei and Harandi, 2010), radon (Lorenzo-González et al., 2019), arsenic (Soza-Ried et al., 2019), chromium (Kouokam et al., 2022), nickel (Shen and Zhang, 1994), uranium (Zhang et al., 2022), vinyl chloride (Girardi et al., 2022) and high dose ionizing radiations (Yan et al., 2022).

## 2 Histopathology

LC is generally divided into four major histological types (adenocarcinoma, squamous cell carcinoma, large cell carcinoma and small cell carcinoma) although combinations may also be present. Among the four histological types, the most represented is adenocarcinoma, accounting for 90%–95% of the LCs and the first three histotypes are collectively known as “Non-Small Cell Lung Carcinoma” (NSCLC).

### 2.1 Non-small cell lung carcinoma (NSCLC)

Adenocarcinoma is the most frequent LC type in women and non-smokers, accounting for 40% of LC (Travis et al., 2011; Ricotti et al., 2021). Unlike squamous cell carcinoma, adenocarcinomas are usually smaller and peripherical in location. Positivity for thyroid transcription factor-1 (TTF-1) is extremely frequent as well as mucin content that is detected in roughly 80% adenocarcinomas. The growing rate is lower compared to squamous cell carcinomas, although adenocarcinomas metastasize earlier and extensively.

Squamous cell carcinomas account for 21% of LC (AIOM, 2019; Ricotti et al., 2021), are more frequent in men and are highly related to smoking habits. From the histological point of view, such histotype is characterized by keratinization.

The main clinical and molecular features of NSCLC are reported in Table 1.

**TABLE 1:** Histological, clinical and molecular features of NSCLC and SCLC. PTH-rp, Parathormone related peptide; ACTH, Adrenocorticotropic hormone; ADH, antidiuretic hormone; GRP, Gastrin releasing peptide.

Histological/Molecular/Clinical feature	NSCLC	SCLC
*Histology*	Abundant cytoplasm; pleomorphic nuclei; prominent nucleoli; gland-like or squamous architecture	Scarce cytoplasm; hyperchromatic nuclei; absent nucleoli; diffused cell layers
*Neuroendocrine markers*	No	Yes
*Epithelial marker*s	Yes	Yes
*Mucin*	Yes (adenocarcinoma)	No
*Peptide hormone production*	Yes (PTH-rp in squamous carcinoma)	Yes (ACTH, ADH, GRP, Calcitonin)

### 2.2 Small cell lung carcinoma (SCLC)

This tumour type accounts for approximately 15% of all lung cancers (Travis et al., 2011; Ettinger et al., 2022) and it is a highly malignant neoplasia characterised by peculiar small round or oval cells with scarce cytoplasm, little or no nucleoli and “salt and pepper” chromatin pattern (Nicholson et al., 2002). Mitotic figures are a frequent finding and necrotic areas are also quite common and extensive. For SCLC, the diagnosis is determined by both light and electron microscopy (to detect neuroendocrine granules) complemented by immunohistochemistry for neuroendocrine markers (chromogranin and synaptophysin) (Righi et al., 2022). The presence of neuroendocrine markers highlights the neuroendocrine origin of SCLC. The main clinical and molecular features of SCLC are reported in Table 1.

SCLC are the most aggressive LC, extensively metastasize, are virtually incurable by surgery and show a close relationship to smoking.

## 3 LC clinical features and treatment

LCs are invasive and silent lesions and represent one of the most insidious and aggressive forms of cancer. LC arises more frequently in people older than 50 years and the main clinical symptoms comprise cough (75%), thoracic pain (40%), weight loss (40%) and dyspnoea (20%).

NSCLC have generally a better prognosis than SCLC. The most important prognostic factor for NSCLC patients is represented by tumour stage and patients are treated according to their stage (Naruke et al., 1997; Schabath and Cote, 2019): surgery is the gold standard for stage I and II patients and for some stage III tumours; stage IV patients are treated with chemotherapy, palliative radiation or supportive therapy.

SCLC are generally diffused also at early stages therefore surgery is not a viable choice for treatment and systemic therapies (such as chemotherapy) are used (Waqar and Morgensztern, 2017).

In addition, in the last decade novel therapeutic targeted agents have been developed thanks to the knowledge of the genetic and molecular alterations carried by LC cells (Table 2).

**TABLE 2:** Genetic alterations in NSCLC and corresponding targeted therapy agents.

Gene	Alteration	Frequency in NSCLC	Targeted therapy
*KRAS*	G12C point mutation	30%	Sotorasib Adagrasib
	G12V point mutation		
	G12D point mutation		
*EGFR*	Exon 19 deletion	15% (Western populations) 35–50% (Asian populations)	Gefitinib
	L858R point mutation		Erlotinib
	L861Q point mutation		Afatinib
	G719X point mutation		Dacomitinib
	T790M point mutation		Osimertinib
*MET*	Mutations	2.5–3%	Capmatinib
	Amplifications		Tepotinib
	Translocations		Crizotinib
			Cabozantinib
*ALK*	EML4-ALK	3–5%	Crizotinib
			Ceritinib
			Alectinib
	KIF5B-ALK		Brigantinib
	KLC1-ALK		Lorlatinib
*ROS1*	CD74-ROS1	0.5–2%	Crizotinib
			Ceritinib
	SDC4-ROS1		Brigatinib
	SLC34A2-ROS1		Lorlatinib
	EZR-ROS1		Entrectinib
			Cabozantinib
*RET*	RET rearrangements	1–2%	Vandetinibv
			Cabozantinib
			Lenvatinib
			BLU-667
*BRAF*	V600E	2.6%	Dabrafenib + Tramafenib
	V469A		
	D594G		
	G466A		
*HER2*	Point mutations	1.8%	Trastuzumab Afatinib
	Amplifications		Ado-trastuzumab Emtansine
*LKB1*	Mutations	8%	Loss of *LKB1* expression is associated with immune check point inhibitor resistance
	Homozygous deletion	30%	
*KEAP1*	Point mutation	15%	Clabetasol propionate
			SW 157765
*NFE2L2*	Point mutation	2%	Clabetasol propionate
			SW 157765

A well-known and frequent side-effect of LC treatment is represented by cardiotoxicity (Pérez-Callejo et al., 2017) and also new strategies such as immune checkpoint or tyrosine kinase inhibitors are associated to cardiac toxic effects, ranging from asymptomatic QT prolongation to acute coronary syndromes, myocardial infarction, reduction in left ventricular ejection fraction, hypertension, symptomatic congestive heart failure and sudden death (Heinzerling et al., 2016). In order to prevent the cardiotoxicity induced by anticancer treatment different adjustments could be applied in the early stages (Cardinale et al., 2016; Pérez-Callejo et al., 2017): healthy lifestyle, modification of anticancer treatment schedules, use of cardioprotective drugs, control of cardiovascular risk factors, periodic evaluation of cardiac function, use of biomarkers in patients with high cardiovascular risk or subjected to highly cardiotoxic anticancer treatment (Pérez-Callejo et al., 2017).

Due to the pivotal role played by certain ion channels (ICs) in heart function, their potential relevance in LC treatment-induced cardiotoxicity has been evaluated (Uchikawa et al., 2022) also because of the modulation exerted by several cytotoxic agents on different ICs (Table 3). For example, crizotinib that has been approved for the NSCLC treatment, inhibits Kv11.1 the ion channel responsible for the delayed-rectifier potassium current in the heart. Such inhibition causes the prolongation of the QT interval in the electrocardiogram, leading to potentially fatal polymorphic ventricular tachycardia, the so-called *torsades de pointes* (Yap and Camm, 2003; Sanguinetti and Tristani-Firouzi, 2006; Shopp et al., 2014; Uchikawa et al., 2022). Interestingly, liposomes administration ameliorates drug-induced effects on Kv11.1 and if they were given prior to crizotinib the effects on the QT interval were decreased (Shopp et al., 2014).

**TABLE 3:** Drugs commonly used for LC treatment affecting ICs.

Drug (LC treatment)	Ion channel affected
*cisplatin*	Kv1.1 Leanza et al. (2014)
	Kv1.3 Leanza et al. (2014)
	Kv1.5 Han et al. (2007)
	Kv10.1 Hui et al. (2015)
	Kv11.1 Zhang et al. (2012), Pillozzi et al. (2017)
	Kir2.1 Liu et al. (2015), Rosa et al. (2018)
	KCa1.1 Samuel et al. (2016)
	STIM1 Gualdani et al. (2019)
	TRPC1 Gualdani et al. (2019)
	Chloride channels Zhang et al. (2018), Okada et al. (2019), Han et al. (2022)
*paclitaxel*	Kv11.1 Chen et al. (2005)
*etoposide*	Kv10.1 Agarwal et al. (2010)
	Kir2.1 Rosa et al. (2018)
*crizotinib*	Kv11.1 Shopp et al. (2014)

## 4 LC genetics

In the last years, several genetic alterations have been described to occur in LC pathogenesis and some of them have been exploited as novel targets for therapy especially in NSCLC (Table 2). Among them, *KRAS* mutations have been shown to occur primarily in the adenocarcinoma histotype at higher frequency in smokers with respect to non-smokers (30% vs. 5%) (Judd et al., 2021). As in other tumours, *KRAS* mutations correlate with worse outcome due to the acquired resistance to epidermal growth factor receptor (*EGFR*) inhibitors (Cascetta et al., 2022). Moreover, mutations and amplifications of the *EGFR* gene are more frequently observed in women, nonsmokers, and people of Asian origin bearing an adenocarcinoma (Herbst et al., 2008). Another gene frequently amplified or mutated in LC is c-*MET* and also in this case targeted therapies have been developed (Herbst et al., 2008). As concerning tumour suppressor genes, the most important (i.e., *Tp53*, *RB1* and *p16*) are inactivated or mutated with similar frequencies in adenocarcinoma and squamous cell carcinoma (roughly 50%, 15% and 65%) (Herbst et al., 2008). Moreover, tumour suppressor genes are also frequently deleted, especially in squamous cell carcinoma and the most frequently involved chromosomes are 3p, 9p, and 17p (Herbst et al., 2008). EGFR is overexpressed in NSCLC and similarly, HER-2/NEU is highly expressed in a small percentage of cases and gene amplification has also been detected (Testa et al., 2018).

SCLC shows a quite high mutation rate related to tobacco carcinogens (Peifer et al., 2012). Several abnormalities have been detected in SCLC, although none of them is specific for this tumour: frequent inactivation of *TP*53, RB1 and PTEN, 3p deletion in the region where the tumour suppressor gene FHIT is located, copy number gain in 7p 22.3, MYC amplification (involving several genes of the MYC family) (Voortman et al., 2010; George et al., 2015), low frequency of activating mutations in KRAS, EGFR and PI3KCA (Testa et al., 2018). Overall, a list of potential driver genes in SCLC has been identified: TP53, RB1, PTEN, SLIT2, EP300, CREBBP, MLL, EPHA7 and COBL (Peifer et al., 2012).

## 5 Ion channels in human cancer

It has been shown more than 30 years ago that cancer cells have a more depolarized membrane potential compared to healthy cells (Binggeli and Weinstein, 1986) and mounting evidences pointed out that ion channels and transporters might represent novel potential biomarkers in human cancers of different histogenesis, since their expression is frequently dysregulated and association with clinico-pathological features and outcome have been shown.

**FIGURE 1:**
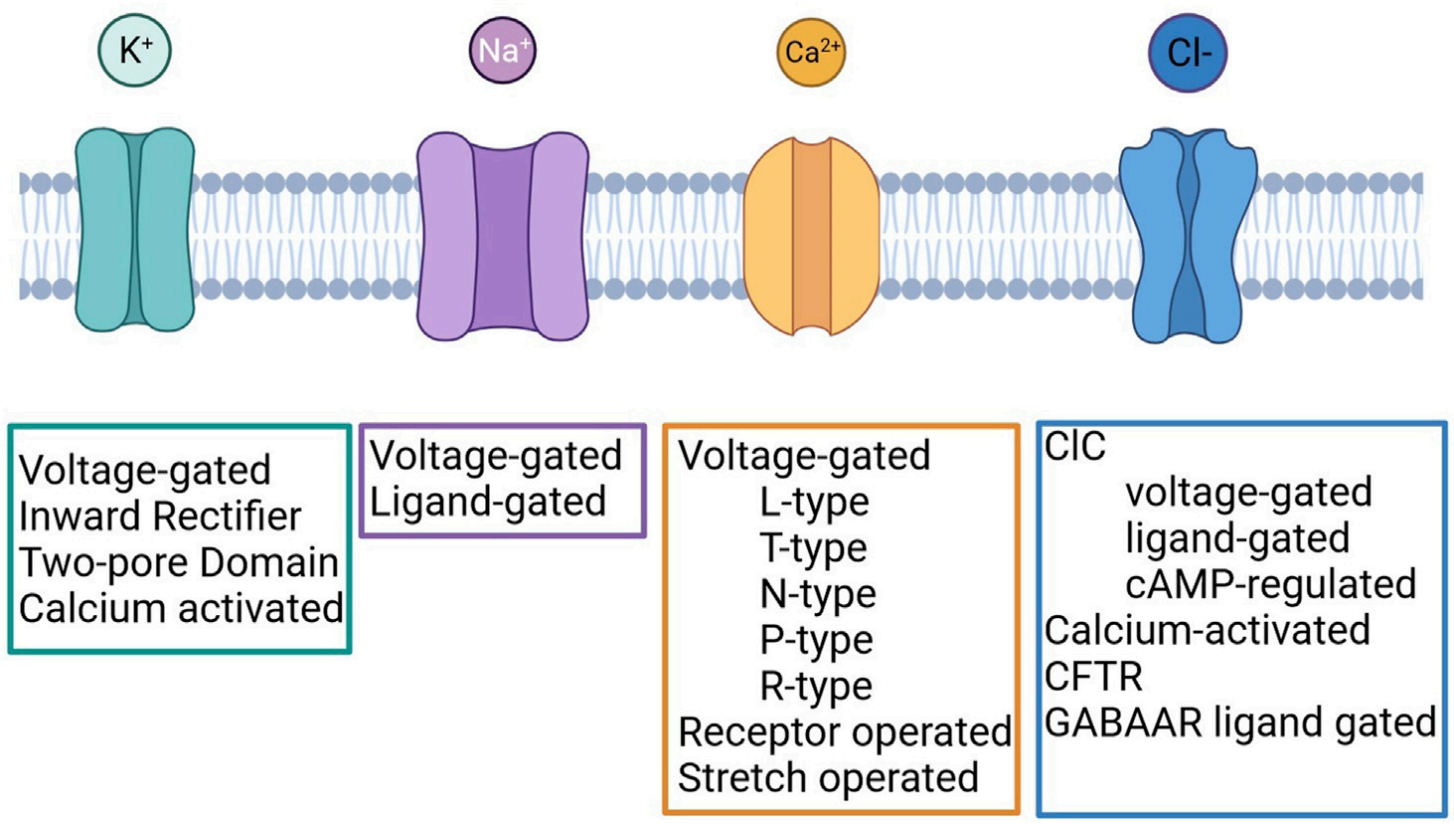
Classification of Potassium, Sodium, Calcium and Chloride channels. Created with BioRender.com.

Several studies carried out in different solid tumours demonstrated that ICs are frequently mis-expressed and play important roles in the regulation of cancer cell behaviour. ICs regulate several cellular processes, and some of them represent hallmarks of cancer (Hanahan and Weinberg, 2000; Hanahan and Weinberg, 2011). The contribute of ICs to cancer development is complex and variegated as the ICs themselves (Figure 1) and the interplay between different members has been described. In fact, the occurrence and functional relevance of ICs within macromolecular complexes occurs in normal and pathological conditions (Heijman and Dobrev, 2018; Ponce-Balbuena et al., 2018; Eichel et al., 2019). It has been shown that in cancer cells ICs interact with different proteins with respect to normal cells (Becchetti et al., 2022). Examples for that are the Kv11.1/β1 integrin complex, selectively expressed in cancer cells (Becchetti et al., 2017), Kv10.1/Orai1/SPCA2 (Badaoui et al., 2018; Peretti et al., 2019), Kv10.1/Calmodulin (Marques-Carvalho et al., 2016), Orai1/TRPC1/SK3 (Potier-Cartereau et al., 2022).

In this context, after proper validation, ICs could represent novel cancer biomarkers (Lastraioli et al., 2015; Anderson et al., 2019). Moreover, since they are located on the cell membranes ICs represent potential targets to be exploited for diagnostic and therapeutic purposes (Arcangeli et al., 2009).

### 5.1 Ion channels in LC

A transcriptomic analysis was carried out to compare the expression of ion channel encoding genes in normal lung tissue and LC (Ko et al., 2014a). Overall, 37 differentially expressed genes were identified: *ANO1, CACNA1C, CACNA1D, CACNA2D2, CACNB3, CLCC1, CLCN3, CLCN7, CLIC3, CLIC4, CLIC5, CLIC6, KCNAB1, KCNAB2, KCNJ2, KCNJ8, KCNE4, KCNK1, KCNK3, KCNK5, KCNQ3, KCNT2, MCOLN1, MCOLN2, MCOLN3, PKD1, PKD2, SCN4B, SCN7A, SCNN1B, SCNN1G, TPCN1, TRPC1, TRPC6, TRPM2, TRPV2, and VDAC1*. It should be pointed out that, although included by Ko and others among the IC encoding genes, some ofthem (namely, *CLCC1, CLCN3, CLCN7, CLIC3, CLIC4, CLIC5, and CLIC6*) are not proper ion channel genes. In order to investigate the prognostic relevance of the above-mentioned genes, a risk score, based on the expression of the differentially expressed genes, was calculated for each patient to predict overall survival and recurrence-free survival in NSCLC. Overall, 31 genes were differentially expressed between adenocarcinoma and squamous cell carcinoma samples (Ko et al., 2014a).

**FIGURE 2:**
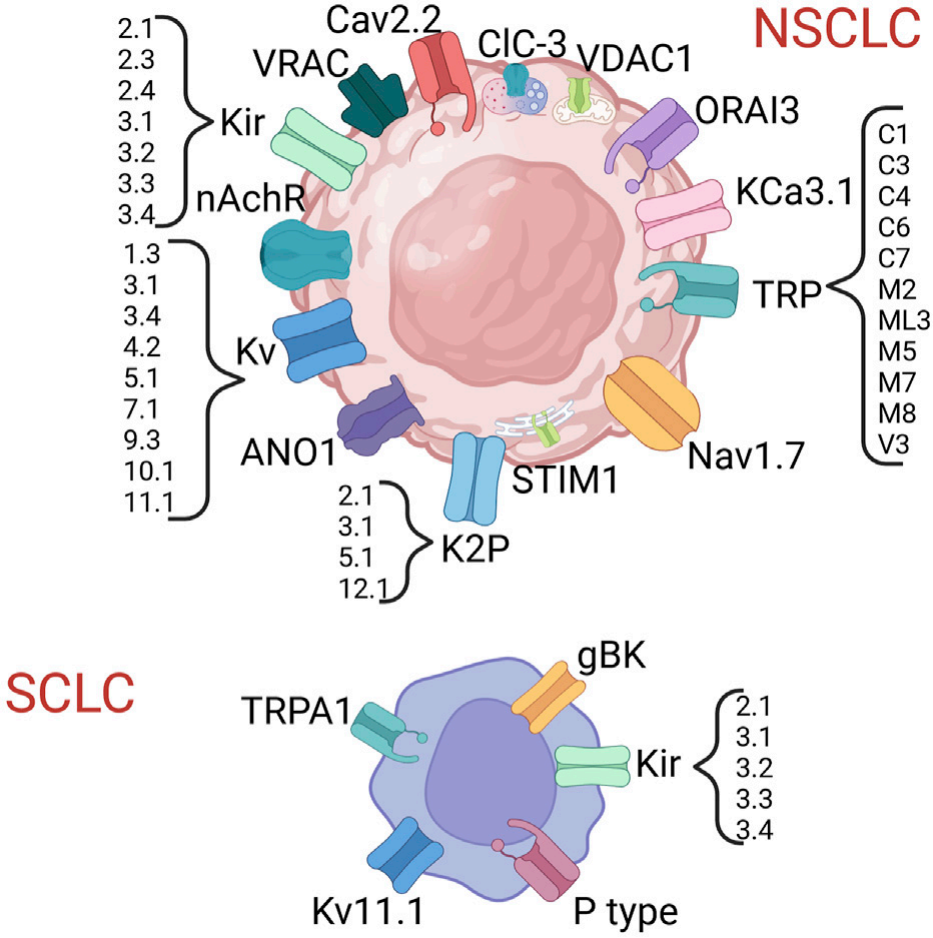
Ion channels shown to be expressed in lung cancer. Created with BioRender.com.

In both NSCLC and SCLC several ICs have been proven to exert a biological role and, in some cases, also to have clinical relevance (Figure 2; Tables 4, 5) [see also the review by Bulk and others (Bulk et al., 2021)].

**TABLE 4:** Ion channels expressed in NSCLC. + = expressed, ++ = overexpressed. EMT, epithelial-mesenchymal transition; TNM, Tumour Node Metastasis.

Channel type	Gene name	Channel name	Expression (cell lines)	Function (cell lines)	Expression (primary tumours)	Clinical correlations
*POTASSIUM*	*KCNN4*	KCa3.1	+ Bulk et al. (2015), Bulk et al. (2017), Bulk et al. (2022); Xu et al. (2021)	Increased expression in more aggressive cells Bulk et al. (2015)		
				Regulation of ICAM-1 dependent cell-cell adhesion between endothelial and cancer cells Bulk et al. (2017)		
				Partial erlotinib resistance can be overcome by channel blockade Glaser et al. (2021)		
				Cell proliferation, migration, invasiveness and tumorigenicity Xu et al. (2021)		
				Regulation of the mitochondria inner membrane potential Bulk et al. (2022)		
	*KCNJ2*	Kir2.1	+ Sakai et al. (2002)	Cell growth and drug resistance Liu et al. (2015)		
	*KCNJ4*	Kir2.3	++ Wu and Yu (2019)		++ Wu and Yu (2019)	Poor prognosis (Wu and Yu, 2019)
	*KCNJ14*	Kir2.4			++ Alasiri (2023)	
	*KCNJ3*	Kir3.1	+ Plummer et al. (2005)		++ Takanami et al. (2004)	Association with lymph node metastasis, stage, negative prognostic factor for overall survival (Takanami et al., 2004)
	*KCNJ6*	Kir3.2	+ Plummer et al. (2005)			
	*KCNJ9*	Kir3.3	+ Plummer et al. (2005)			
	*KCNJ5*	Kir3.4	+ Plummer et al. (2005)			
	*KCNA3*	Kv1.3	+ Jang et al. (2011a)	Cell proliferation Jang et al. (2011a)	+ Angi et al. (2023)	Decrease with tumour stage progression, associates with patient prognosis (Angi et al., 2023)
	*KCNC1*	Kv3.1	+ Song et al. (2018)	Cell migration and invasiveness Song et al. (2018)		
	*KCNC4*	Kv3.4	+ Song et al. (2018)	Cell migration and invasiveness Song et al. (2018)		
	*KCND2*	Kv4.2			++ Lu et al. (2021b)	Poor prognosis (Lu et al., 2021b)
	*KCNF1*	Kv5.1	+ Chen et al. (2023)	Cell proliferation Chen et al. (2023)		
	*KCNQ1*	Kv7.1	+ Girault et al. (2014), Chang et al. (2022)	Cell proliferation and migration Girault et al. (2014)	++Girault et al. (2014), Chang et al. (2022)	Potential target for therapeutic intervention (Girault et al., 2014)
						Independent Risk Factor (Chang et al., 2022)
	*KCNS3*	Kv9.3	+ Lee et al. (2015)	Cell proliferation Lee et al. (2015)		
				Cell cycle Song et al. (2018)		
	*KCNH1*	Kv10.1	+ Hemmerlein et al. (2006), Restrepo-Angulo et al. (2011), Acuña-Macías et al. (2015)	Upregulation during EMT Restrepo-Angulo et al. (2011) Cell proliferation Acuña-Macías et al. (2015)		
	*KCNH2*	Kv11.1	+ Glassmeier et al. (2012)	Cell proliferation Glassmeier et al. (2012)		
	*KCNK2*	K_2P_2.1			++ Williams et al. (2013)	
	*KCNK3*	K_2P_3.1	− Lin et al. (2022)	Negative regulator of cell proliferation Lin et al. (2022)	- Lin et al. (2022)	Decreased expression correlated with poor prognosis (Lin et al., 2022)
	*KCNK5*	K_2P_5.1			++ Williams et al. (2013)	
	*KCNK12*	K_2P_12.1			++ Williams et al. (2013)	
	*KCNAB2*	HKvbeta2	− Lyu et al. (2022)		− Lyu et al. (2022)	Decreased and associated with poor prognosis, reduced immune infiltration (Lyu et al., 2022)
*SODIUM*	*SCN9A*	Nav1.7	++ Campbell et al. (2013)	Cell invasiveness Campbell et al. (2013)	++ Campbell et al. (2013)	Potential target for therapeutic intervention and/or as a diagnostic or prognostic marker (Campbell et al., 2013)
*CALCIUM*	*TRPC1*	TRPC1	+ Gualdani et al. (2019)	Cisplatin toxicity Gualdani et al. (2019)	+ Jiang et al. (2011), Jiang et al. (2013)	Differentiation (Jiang et al., 2011)
	*TRPC3*	TRPC3			+ Jiang et al. (2011)	Differentiation (Jiang et al., 2011)
	*TRPC4*	TRPC4			+ Jiang et al. (2011)	Differentiation (Jiang et al., 2011)
					SNPs Zhang et al. (2016)	Increased risk (Zhang et al., 2016)
	*TRPC6*	TRPC6	+ Yang et al. (2017)	Cell proliferation Yang et al. (2017)	+ Jiang et al. (2011)	Differentiation (Jiang et al., 2011)
	*TRPC7*	TRPC7			SNPs Zhang et al. (2016)	Increased risk (Zhang et al., 2016)
	*TRPM2*	TRPM2	+ Almasi et al. (2019)	Cell proliferation, apoptosis, cell invasiveness Almasi et al. (2019)		
	*MCOLN3*	TRPML3	+ Kim et al. (2022)	Drug resistance Kim et al. (2022)		
	*TRPM5*	TRPM5	+ Huang et al. (2017)	Migration Huang et al. (2017)		
	*TRPM7*	TRPM7	+ Chen et al. (2014)	Migration Chen et al. (2014)		
	*TRPM8*	TRPM8	+ Du et al. (2014)	Migration Du et al. (2014)		
	*TRPV3*	TRPV3			++ Li et al. (2016)	Tumour progression, companion drug target Li et al. (2016)
	*CACNA1B*	Cav2.2			+ Zhou et al. (2017)	TNM, progression Zhou et al. (2017)
	*ORAI 3*	ORAI 3	+ Ay et al. (2013)	Cell proliferation Ay et al. (2013)	++ Ay et al. (2013)	High grade Ay et al. (2013)
				Chemoresistance induction in CSC Daya et al. (2021)		
	*STIM1*	STIM1	+ Gualdani et al. (2019)	Cisplatin toxicity Gualdani et al. (2019)		
*CHLORIDE*	*CLCN3*	ClC-3	+ Chen et al. (2019)	Drug resistance Chen et al. (2019)		
	*CLIC1*	CLIC1	+ Lee et al. (2019)	Cell survival		
	*LRRC8*	VRAC	+ (He et al., 2010)	Carboplatin-induced apoptosis He et al. (2010)		
*ANIONS*	*ANO1*	ANO1	+ Jia et al. (2015), Seo et al. (2021)	Cell proliferation and invasiveness Jia et al. (2015)	++ Jia et al. (2015)	Potential target for therapeutic intervention Jia et al. (2015)
				Potential target for therapeutic intervention Seo et al. (2021), Jeong et al. (2022)		
	*VDAC1*	VDAC1	+ Zhang et al. (2020)	Potential target for therapeutic intervention Zhang et al. (2020)	+ Grills et al. (2011), Ko et al. (2014b)	Negative prognostic factor Grills et al. (2011) also included in a specific signature Ko et al. (2014b)
*TRANSPORTERS*	*CHRNA5*	α5-nAChR	+ Ma et al. (2014)	Potential target for therapeutic intervention Ma et al. (2014)	+ Falvella et al. (2009)	p.Asp398Asn polymorphism in the CHRNA5 gene is associated with LC risk Falvella et al. (2010)
	*CHRNA7*	α7-nAChR	+ Ma et al. (2014)	Cell proliferation, migration and invasiveness Ma et al. (2014)	++ Ma et al. (2019)	Negative prognostic factor Ma et al. (2019)

**TABLE 5:** Ion channels expressed in SCLC. + = expressed, ++ = overexpressed.

Channel type	Gene name	Channel name	Expression (cell lines)	Function (cell lines)	Expression (primary tumors)	Clinical correlations
*POTASSIUM*	*KCNJ2*	Kir2.1	+ Liu et al. (2015)	Cell proliferation and MDR modulation Liu et al. (2015)	++ Liu et al. (2015)	Correlation with stage and response to chemotherapy, prognostic factor Liu et al. (2015)
	*KCNJ3*	Kir3.1	+ Plummer et al. (2005)			
	*KCNJ6*	Kir3.2	+ Plummer et al. (2005)			
	*KCNJ9*	Kir3.3	+ Plummer et al. (2005)			
	*KCNJ5*	Kir3.4	+ Plummer et al. (2005)			
	*KCNH2*	Kv11.1	+ Glassmeier et al. (2012)	Cell proliferation Glassmeier et al. (2012)		
	*KCNMA1*	gBK			++ Hoa et al. (2014)	Late-stage marker Hoa et al. (2014)
*CALCIUM*	*TRPA1*	TRPA1	+ Schaefer et al. (2013)	Cell survival Schaefer et al. (2013)		
	*CACNA1A*	P-type	+ Barry et al. (1995)			

#### 5.1.1 Ion channels in lung carcinogenesis

**FIGURE 3:**
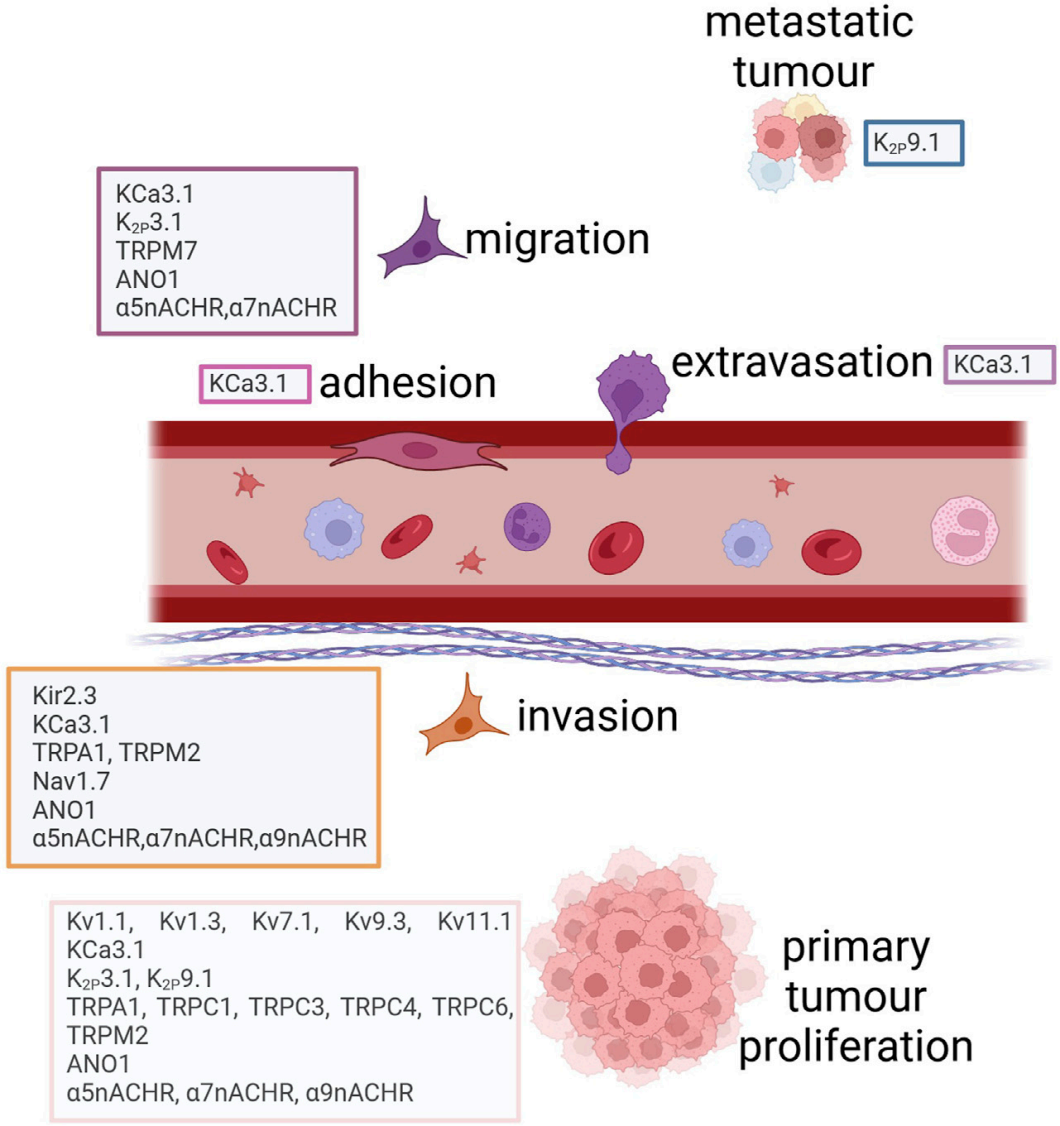
Ion channels involved in lung cancer progression. Created with BioRender.com.

Lu and others performed an *in silico* analysis showing that six ion channel genes (*GJB2, CACNA1D*, *KCNQ1, SCNN1B, SCNN1G,* and *TRPV6*) were differentially expressed in lung tumorigenesis (Lu A. et al., 2021). Among the six genes, lower expression of *SCNN1B* (through the hypermethylation of the promoter region) was associated with shorter overall survival. Also, the inactivation of FXYD3, an IC-related protein, was proven long ago to play a role in LC progression (Okudela et al., 2009). Among TRP channels, it was demonstrated that TRPM7 mRNA and protein levels are regulated by EGF, an activator of migration in LC (Gao et al., 2011). In general, ICs modulate different processes within the LC cell (proliferation, migration, invasiveness, drug resistance) thus their expression appears to be relevant in all the phases of LC carcinogenesis (Tables 4, 5). A scheme showing the main ICs involved in the different cell processes is reported in Figure 3.

In some cases, ICs are also regulated by LC risk factors. For example, nicotinic acetylcholine receptors are activated by compounds present in tobacco, such as nicotine and 4-(methylnitrosamino)-1-(3-pyridyl)-1-butanone (Grando, 2014) (see also paragraph 5.1.6). Similarly, miners are exposed to radon that represents a known risk factor for LC and it was demonstrated through a genome wide association study (GWAS) that six markers within the *CHRNA5* and *CHRNB4* genes, encoding the nicotinic cholinergic receptor alpha 5 and beta 4 subunits respectively, are associated with higher LC risk (Rosenberger et al., 2018). Arsenic, another well-known LC risk factor, modulates several potassium channels, namely, KCNA5 (positively associated with arsenic levels), CACNA1, KCNH2, KCNQ1, and KCNE1 (downregulated by arsenic) (Mo et al., 2011). Ionizing radiations are also an example of a LC risk factor that is associated with ICs. In particular, it was demonstrated that TRPM2 and TRPV1 channels are involved in the responses to γ- and UVB- irradiation DNA damage (Masumoto et al., 2013).

#### 5.1.2 Potassium channels in LC

Recently, an *in silico* study addressing the clinical relevance of potassium channels in LC was published (Ko et al., 2019). The Authors identified 10 deregulated genes (5 up- and 5 downregulated) comparing LC and healthy lung tissue. A risk scoring was defined taking into account this 10 gene signature in order to predict clinical outcome independently from standard clinical prognostic factors, that might therefore be used along with conventional factors.

Potassium channels are a multi-gene family composed of four different subfamilies: voltage-gated potassium channels (VGKCs), inward rectifiers (IRK), two-pore domains (K_2P_) and calcium-activated channels (KCa) (Figure 1). In LC, several members of each subfamily have been detected and their role was investigated.

Among VGKC, different members (Kv1.3, Kv3.1, Kv3.4, Kv4.2, Kv5.1, Kv7.1, Kv9.3, Kv10.1, and Kv11.1) are involved in LC cell proliferation, migration and invasion (Jang et al., 2011a; Restrepo-Angulo et al., 2011; Glassmeier et al., 2012; Girault et al., 2014; Acuña-Macías et al., 2015; Lee et al., 2015; Chen et al., 2023) and some of them (Kv1.3, Kv4.2, and Kv7.1) have clinical relevance in NSCLC (Girault et al., 2014; Lu X. et al., 2021; Chang et al., 2022; Angi et al., 2023) (Table 4).

Kv11.1 channels are expressed in SCLC cell lines and they were shown to regulate cell proliferation (Glassmeier et al., 2012). Glioma Big Potassium Channels (gBK) are expressed in advanced SCLC thus representing a late-stage marker for this condition (Hoa et al., 2014) (Table 5).

Members of the IRK family were also shown to be involved in LC. In particular, Kir2.1, Kir2.4, Kir3.2, Kir3.3, and Kir3.4 are expressed in NSCLC (Sakai et al., 2002; Plummer et al., 2005; Wu and Yu, 2019) and SCLC (Plummer et al., 2005; Liu et al., 2015) cell lines (Tables 4, 5). Other members of the same family (Kir2.3 and Kir2.4) are expressed in primary LC (Takanami et al., 2004; Wu and Yu, 2019; Alasiri, 2023) and are associated with poor prognosis (Takanami et al., 2004; Wu and Yu, 2019) and lymph node involvement (Takanami et al., 2004) (Tables 4, 5). Interestingly, Kir2.1 regulates cell proliferation and multidrug resistance (MDR) in both NSCLC and SCLC cells (Liu et al., 2015; Wu and Yu, 2019) and also represents a prognostic factor, being overexpressed in primary SCLC and correlating with stage and response to chemotherapy (Liu et al., 2015) (Tables 4, 5).

The K_2P_ subfamily is also involved in LC pathogenesis and it was shown that K_2P_3.1 is downregulated in NSCLC cells (where it negatively regulates cell proliferation) and in primary tumours where it is associated with poor prognosis (Lin et al., 2022). On the other hand, K_2P_2.1, K_2P_5.1, and K_2P_12.1 are over-expressed in primary NSCLC (Williams et al., 2013) (Table 4).

The only member of the Calcium activated subfamily that has been shown to be expressed in NSCLC is KCa3.1 (Bulk et al., 2015; 2017; 2022; Xu et al., 2021) (Table 4). In particular, increased expression of the channel was detected in more aggressive cells (Bulk et al., 2015) where it also regulates ICAM-1 dependent cell-cell adhesion between endothelial and cancer cells (Bulk et al., 2017). Moreover, KCa3.1 regulates cell proliferation, migration, invasiveness and tumorigenicity (Xu et al., 2021) and the mitochondria inner membrane potential (Bulk et al., 2022) (Table 4). Interestingly, it was shown that blocking the channel results in partial overcome of erlotinib resistance (Glaser et al., 2021) (Table 4).

#### 5.1.3 Sodium channels in LC

Voltage Gated Sodium Channels of the Voltage-gated subfamily (Figure 1) have been shown to be expressed in NSCLC cells, where they possibly regulate tumour cell invasiveness, as in other tumour types (Onganer and Djamgoz, 2005; Fraser et al., 2014; Nelson et al., 2014; Djamgoz et al., 2019; Haworth and Brackenbury, 2019). Interestingly, the Nav blocker tetrodotoxin reduces the invasiveness of NSCLC cell lines (Roger et al., 2007; Campbell et al., 2013). In addition, the role of Nav1.7 channel, encoded by *SCN9A* gene, was investigated (Campbell et al., 2013). The Authors showed an EGFR-mediated transcriptional regulation of the channel expression, responsible of the invasive behaviour of NSCLC cells. The administration of the EGFR blocker gefitinib also affects Nav1.7 at the mRNA and protein level, as well as the sodium current (Campbell et al., 2013). Moreover, the immunohistochemical evaluation of primary samples suggested that Nav1.7 expression could have clinical relevance in NSCLC.

#### 5.1.4 Calcium channels in LC

The expression of both voltage-gated and ligand-gated calcium channels (Figure 1) has been described in LC (Tables 4, 5). The most represented are TRP (Transient Receptor Potential) channels. In particular, TRPC1, TRPC6 TRPM2, TRPML3, TRPM5, TRPM7, and TRPM8 were found to be expressed in NSCLC cells (Chen et al., 2014; Du et al., 2014; Huang et al., 2017; Yang et al., 2017; Almasi et al., 2019; Gualdani et al., 2019; Kim et al., 2022). TRPC1 mediates cisplatin toxicity (Gualdani et al., 2019), TRPC6 and TRPM2 are involved in cell proliferation (Yang et al., 2017; Almasi et al., 2019), TRPM5, TRPM7 and TRPM8 modulate cell migration (Chen et al., 2014; Du et al., 2014; Huang et al., 2017) and TRPML3 is involved in MDR (Kim et al., 2022).

Members of the TRP family are also expressed in primary NSCLC: TRPC3 and TRPC6 (associated with differentiation) (Jiang et al., 2011), TRPC4 and TRPC7 (whose single nucleotide polymorphisms, SNPs) are associated with increased risk of LC) (Zhang et al., 2016). TRPV3 are overexpressed in primary NSCLC and have been proposed as drug companion (Li et al., 2016). In SCLC TRPA1 plays a pivotal role in cell survival (Schaefer et al., 2013) (Table 5).

The only member of the voltage-gated subfamily that has been detected in NSCLC is Cav2.2, overexpressed in primary tumours and associated with TNM stage and tumour progression (Zhou et al., 2017). In SCLC cell lines P-type calcium channels have been detected (Barry et al., 1995) (Table 5).

Two members of the Store-Operated subfamily of ligand-gated channels (ORAI 3 and STIM1) were also found to be expressed in NSCLC cells regulating cell proliferation (ORAI 3) (Ay et al., 2013) and cisplatin toxicity (STIM1) (Gualdani et al., 2019) (Table 4). Moreover, ORAI3 is involved in the chemoresistance induction of Cancer Stem Cells (CSC) (Daya et al., 2021) and is associated with high grade primary lesions (Ay et al., 2013).

#### 5.1.5 Chloride and anion channels in LC

Among Chloride channels (Figure 1), three members have been shown to be expressed in NSCLC cells: the Chloride Voltage-gated channel 3 (ClC-3), functioning as a Chloride-Hydrogen antiporter (associated to MDR) (Chen et al., 2019), CLIC1 (that mediates cell survival) (Lee et al., 2019) and VRAC (involved in Carboplatin-induced apoptosis) (He et al., 2010) (Table 4).

The voltage-gated calcium-activated anion channel Anoctamin-1 (ANO1) is expressed in both NSCLC cell lines and primary tumours, mediating cell proliferation and invasiveness (Jia et al., 2015) and representing a potential therapeutic target (Jia et al., 2015; Seo et al., 2021; Jeong et al., 2022).

The voltage-dependent anion channel type 1 (VDAC1) was found to be expressed in NSCLC cells and was proposed as a therapeutic target (Zhang et al., 2020). Accordingly, a meta-analysis of surgically resected NSCLC led to identify VDAC1 as one of the most relevant genes. In particular, the channel was associated with poor overall survival and was an independent prognostic factor (Grills et al., 2011). Moreover, VDAC1 expression levels were upregulated in tumours compared with normal tissue including lung (Ko et al., 2014b). 44 VDAC1 interacting genes were identified and included (along with VDAC1) into a gene signature that turned out to be a prognostic biomarker to predict recurrence-free survival (Ko et al., 2014b).

#### 5.1.6 Nicotinic acetylcholine receptors in LC

The nicotinic acetylcholine receptors (nAChRs) are the most studied channel type in LC (Table 4) (Falvella et al., 2009) and their relevance in LC carcinogenesis was hypothesized long ago, since they are activated by compounds present in tobacco, such as nicotine and 4-(methylnitrosamino)-1-(3-pyridyl)-1-butanone (Grando, 2014). nAChRs are a heterogeneous IC family comprising α and β subunits expressed in neurons, bronchial cells and keratinocytes (Egleton et al., 2008).

GWAS showed that the 15q25 nAChR gene cluster *CHRNA5-A3-B4* is associated with nicotine dependence and LC (Amos et al., 2008). Moreover, the expression of the *CHRNA5* gene encoding α5-nAchR was found to be increased in LC tissue and the p.Asp398Asn polymorphism was associated with LC risk (Falvella et al., 2010). Interestingly, the expression of α5-nAchR correlates with the hypoxia inducible factor (HIF) 1α in NSCLC (Ma et al., 2014) and since a α5-nAChR/HIF-1α/VEGF axis is involved in nicotine-induced tumor cell proliferation, α5-nAChR might represent a potential anticancer target in LC (Ma et al., 2014). The activation of nAChR by nicotine increases the migration and invasiveness of A549 LC cells (Sun and Ma, 2015) while silencing reverses the process (Zhang et al., 2017). The α7-nAChR subunit is upregulated in NSCLC tissue samples (Ma et al., 2019) and an *in silico* analysis indicated that this correlates with poor outcome.

### 5.2 Ion channels as therapeutic targets

As previously stated, LC treatment nowadays can benefit from molecular targeting (including the application of small molecule tyrosine kinase inhibitors and monoclonal antibodies) that is generally well tolerated by patients (Bittner et al., 2014). Since ICs have been proven to play major roles in LC pathogenesis and progression their relevance as potential therapeutic targets was also evaluated. Several drugs acting as IC blocker exist and some of them have also been tested in clinical trials for other malignancies. For example, the opioid U50488H was shown to promote chemosensitivity, to inhibit proliferation and growth of NSCLC (Kuzumaki et al., 2012) and to block Kir3.1–4 channels (Plummer et al., 2005; D’Amico et al., 2013). Senicapoc, an inhibitor of KCa3.1 channels, was used in a phase III clinical trial for sickle cell anemia (Ataga et al., 2011), and it was also shown to reduce tumor growth in mice xenografted with A549 NSCLC cells (Bulk et al., 2015). The same occurs for another potassium channel blocker (dendrotoxin-κ) acting on Kv11.1 (Jang et al., 2011b). Margatoxin, an inhibitor of Kv1.3 potassium channel, exerts an antiproliferative effect on A549 cells (Jang et al., 2011a). The TRPV6 inhibitor SOR-C13 was used in a phase I clinical trial for the treatment of advanced solid tumors (Fu et al., 2017). IC inhibitors have also been used in combination therapy with chemotherapeutic agents as the Cav3.1 channel blocker in A549 cells (Byun et al., 2016). This aspect is particularly relevant since a combined therapy might overcome the resistance of tyrosine kinase inhibitors as gefitinib (Jeon et al., 2012), or reduce chemotherapy side effects. On the other hand, a general problem to be faced when using K^+^ channel blockers is represented by their relevance and role in excitable cells (Arcangeli and Becchetti, 2010). An example is represented by Kv11.1 that plays a pivotal role in the repolarization phase following the action potential in cardiomyocytes (Sanguinetti and Tristani-Firouzi, 2006). For this reason, blocking Kv11.1 causes the prolongation of the QT interval thus leading to ventricular arrhythmia and fibrillation with the generation of *torsade de pointes* (Witchel and Hancox, 2000). The main Kv11.1 blockers belong to class III antiarrhythmic drugs, nevertheless the channel is also blocked by other types of compounds, such as antibiotics (erythromycin), antihistaminics (terfenadine), antipsychotics (sertindole) and prokinetics (cisapride). Importantly, not all Kv11.1 blockers are arrhythmogenic (Wallis, 2010), examples are represented by Verapamil and sertindole (D’Amico et al., 2013). In order to overcome the cardiotoxicity induced by IC blockers different strategies can be applied (D’Amico et al., 2013): i) using non torsadogenic blockers; ii) using state-specific blockers such as *R*-roscovitine (Ganapathi et al., 2009); iii) using tumour-specific drugs such as CD 160130 (Gasparoli et al., 2015); iv) using monoclonal antibodies (Iorio et al., 2019) and v) using bispecific antibodies directed against tumour-specific macromolecular complexes such as the Kv11.1/β1 integrin complex (Duranti et al., 2021a; 2021b; Iorio et al., 2022; Lottini et al., 2023).

## 6 Concluding remarks

Lung cancer is an important health issue worldwide due to the high incidence and mortality, especially for SCLC, thus, searching for novel biomarkers and targets for LC is mandatory.

**FIGURE 4:**
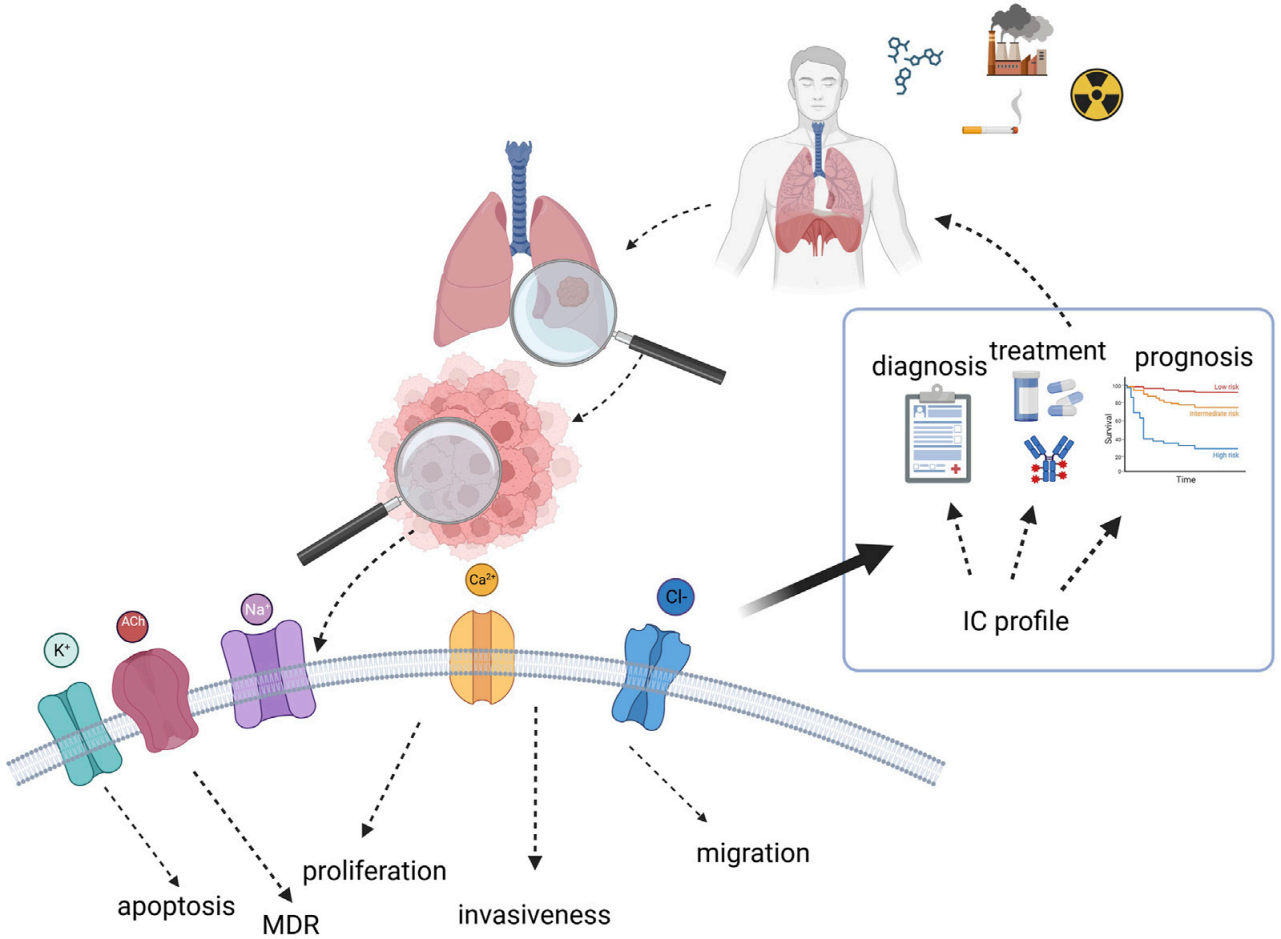
Cartoon summarizing the role and relevance of ion channels in lung cancer and their possible exploitation for clinical purposes. Created with BioRender.com.

It is now known that ICs control several cancer hallmarks and could therefore serve as molecular markers in cancer. Moreover, blocking the activity of ICs impairs tumour growth, paving the road to the pharmaceutical exploitation of ICs. Due to their peculiar localization, ICs can be also easily detected and blocked by either drugs or antibodies. In the context of LC, the relevance of ICs is multiple (see the cartoon depicted in Figure 4) since some of them are activated by known risk factors and might be the molecular cause of cardiotoxicity induced by therapeutic agents commonly used in LC; on the other hand, ICs modulate key cell processes and contribute to LC progression. Finally, ICs might be exploited for diagnostic and prognostic purposes, as well as being used as novel therapeutic targets.
